# Developing an Asthma Self-management Intervention Through a Web-Based Design Workshop for People With Limited Health Literacy: User-Centered Design Approach

**DOI:** 10.2196/26434

**Published:** 2021-09-09

**Authors:** Hani Salim, Ping Yein Lee, Sazlina Sharif-Ghazali, Ai Theng Cheong, Jasmine Wong, Ingrid Young, Hilary Pinnock, RESPIRE Collaboration

**Affiliations:** 1 NIHR Global Health Research Unit on Respiratory Health (RESPIRE) Usher Institute The University of Edinburgh Edinburgh United Kingdom; 2 Department of Family Medicine Medical Faculty and Health Sciences Universiti Putra Malaysia Serdang Malaysia; 3 UM eHealth Unit Faculty of Medicine University Malaya Petaling Jaya Malaysia; 4 Malaysian Research Institute on Ageing Universiti Putra Malaysia Serdang Malaysia; 5 Centre for Biomedicine, Self and Society Usher Institute University of Edinburgh Edinburgh United Kingdom

**Keywords:** asthma, self-management, design sprint, health literacy, mobile phone

## Abstract

**Background:**

Technology, including mobile apps, has the potential to support self-management of long-term conditions and can be tailored to enhance adoption. We developed an app to support asthma self-management among people with limited health literacy in a web-based workshop (to ensure physical distancing during the COVID-19 pandemic).

**Objective:**

The aim of this study is to develop and test a prototype asthma self-management mobile app tailored to the needs of people with limited health literacy through a web-based workshop.

**Methods:**

We recruited participants from a primary care center in Malaysia. We adapted a design sprint methodology to a web-based workshop in five stages over 1 week. Patients with asthma and limited health literacy provided insights into real-life self-management issues in stage 1, which informed mobile app development in stages 2-4. We recruited additional patients to test the prototype in stage 5 using a qualitative research design. Participants gave feedback through a concurrent thinking-aloud process moderated by a researcher. Each interview lasted approximately 1 hour. Screen recordings of app browsing activities were performed. Interviews were audio-recorded and analyzed using a thematic approach to identify utility and usability issues.

**Results:**

The stakeholder discussion identified four themes: individual, family, friends, and society and system levels. Five patients tested the prototype. Participants described 4 ways in which the app influenced or supported self-management (utility): offering information, providing access to an asthma action plan, motivating control of asthma through support for medication adherence, and supporting behavior change through a reward system. Specific usability issues addressed navigation, comprehension, and layout.

**Conclusions:**

This study proved that it was possible to adapt the design sprint workshop to a web-based format with the added advantage that it allowed the development and the testing process to be done efficiently through various programs. The resultant app incorporated advice from stakeholders, including sources for information about asthma, medication and appointment reminders, accessible asthma action plans, and sources for social support. The app is now ready to move to feasibility testing.

## Introduction

### Background

Supported self-management for asthma (written action plans and regular review) is highly effective at improving control and reducing acute attacks [1-3]; however, globally, it is challenging to implement for 334 million people living with asthma [4-7]. One of the challenges is the need to tailor support for people with limited health literacy. Health literacy is defined as the degree to which individuals can obtain, process, and understand the necessary health information needed to make appropriate health decisions [8]. Studies have associated limited health literacy with erroneous health beliefs and poor adherence to self-management activities [9,10]. Malaysia has a high burden of limited health literacy in the general population [11], and asthma control is challenged by a lack of patient education, overreliance on unscheduled visits, and lack of action plan ownership [12-14].

The use of digital technologies for internet-based information is more common in the younger age group than in the middle and older age groups [15]. Malaysia’s multigenerational household culture and strong family orientation have helped younger family members assist older generations in using digital technology to stay connected and find information [16]. The pandemic has further seen widespread adoption of digital technologies by a broader age group of users in diagnosis, prevention, and surveillance [17]. Three-fourth of Malaysians are now smartphone users, with most (60.9%) of them in the lowest income group [18]. Although health-related information-seeking behavior on the internet is greater in those with good health literacy [19], our previous qualitative work among people with limited health literacy suggests that a mobile app is a preferred medium to deliver supported self-management, including a pictogram-based asthma action plan and signposting to reliable asthma information sources.

### Goals

For the aforementioned reasons, developing asthma self-management tailored to limited health literacy needs is an important context for the web-based design sprint workshop, as various studies have shown that the extensive use of pictograms, images, and prompts was appealing to participants and may improve the understanding of information in mobile apps [20-23]. However, it is essential to involve users early in the design stage, as some of the unique features that people want can be time-consuming and costly to build, and a balance may need to be found between desired features [24] and those with evidence-based recommendations [25,26].

Using a design sprint methodology, we seek to optimize user experience in app development by integrating patients into the 5-stage process of mapping, sketching, designing, developing, and testing [24,27]. We used the health literacy framework [8] to underpin the overall structure of the interventional work. The COVID-19 pandemic and physical distancing requirements meant that we had to conduct our workshop in a web-based format. In this study, we report the outcomes of the workshop deliberations and our experience of conducting a week-long remote 5-stage program attended by patients with asthma and health care professionals (HCPs).

## Methods

### Ethical Consideration

The workshop received ethical approval from the Medical Research and Ethics Committee of the Ministry of Health, Malaysia (ID: NMRR-19-3609-52292) and sponsorship approval by the Academic and Clinical Central Office for Research & Development at the University of Edinburgh (ID: AC20011). Informed consent was obtained from all the participants before the workshop.

### Study Design

We conducted a 5-stage design sprint workshop using a web-based and qualitative research approach. We used the 5-stage design sprint process as a roadmap to develop the intervention. We adapted the methodology and constructed the workshop into five stages: (1) understanding and mapping problems, (2) sketching of solutions, (3) deciding on solutions to problems, (4) developing a prototype, and (5) testing a low-fidelity prototype [27,28]. The process was originally designed in the technology sector by the Google Ventures team for business start-up teams [27]. The involvement of the target population and early testing enhance intervention effectiveness and increase the likelihood of adoption at the implementation stage [24]. Owing to the rapid development and testing stages, this is an ideal concept for a low-resource setting, that is, time and cost [24].

Stakeholder (patient and HCP) discussions provided insights into self-management issues in stage 1, which informed mobile app development in stages 2-4. We recruited patients with asthma and limited health literacy to test the low-fidelity prototype in stage 5 and provided feedback through qualitative interviews. A low-fidelity prototype is a modeled prototype with limited technical functionality [29] that is quick to create and can be easily improved in the light of feedback in the testing stages. The details of each stage are listed in Table 1.

**Table 1:** Process, outcomes, and web-based adaptations of the workshop.

Stages and objectives			Process		Adaptations for web-based delivery of the workshop
**Stage 1: understand and mapping problem**					
	To identify the objectives of the prototype and the workshop To map out problems from health care professionals and patients’ perspectives which technology can help to solve	As a team, we first discussed and agreed on the workshop’s long-term goals for the workshop’s prototype and aims through a structured discussion between the patients and health care professionals. We listed a list of problems relating to self-managing asthma from stakeholders (patients and clinicians) point of views. We constructed an end-to-end process of how patients cared for their asthma, and we targeted the problems we could potentially provide solutions for using the mobile app.		Through a web conference site (Microsoft Teams), we brainstormed the long-term goal for the app and the workshop’s aim. In a separate browser, using a web-based board (Miro), we gathered the problems, potential solutions, and mapping of the target where the solutions can occur. Interviews were audio recorded during the workshop. We considered scientific literature and previous study we have conducted as expert input.	
**Stage 2: sketch solution**					
	To understand a broad range of problems and solutions concerning asthma self-management	Focusing on the problems, each researcher reviewed existing ideas which we could potentially use and improve for the prototype. The individual researcher then presented their findings and the reasons why the ideas being chosen. Using this information, we then drew crude scenes with our contents which we believed would be suitable for the app. After presenting the scenes and critical discussions, we voted on the best scene and content for the prototype.		Reviewing and compiling sketches of ideas were done on Miro synchronously by all researchers. We presented these sketches of ideas to the whole team on Microsoft Teams. Individually, using colorful sticky notes and marker pens, we drew the crude scenes. We took photographs of these scenes and uploaded these on Miro. Each researcher was given three blue dots for the voting, and they placed a dot on the best ideas.	
**Stage 3: decide the solution for the problem**					
	To decide on solutions that answered our long-term objectives	The winning scenes and content comprised topics on asthma education, asthma symptoms monitoring, and supporting people living with asthma. We took the winning scenes from our sketches, and we constructed an end-to-end process (storyboard) on how these scenes and content would appear on an App. The storyboard was first constructed in text form before we transformed it visually.		The most voted ideas were put together, and we had another round of voting where each researcher was given a pink dot, and the team leader was given three purple dots on the web-based board (Miro). The text and visual version of the storyboard was constructed as a group and through discussions on the web-based board (Miro) and Microsoft Teams.	
**Stage 4: prototype development**					
	To build ideas for a low-fidelity prototype	Each of the research members and the App developer was assigned roles to ensure the successful development of the low-fidelity prototype for the final day testing process.		On Figma, a prototype development site, the low-fidelity prototype was developed. Every researcher and the App developer completed their tasks (ie, content and language check) within Figma. Brainstorming of prompts for the testing day was conducted on Google Sheets among the researchers. Google sheet also was where a virtual scoreboard was set up for every researcher to capture the patient’s evaluation of the prototype.	
**Stage 5: test low-fidelity prototype**					
	To validate the solutions for the patients through a qualitative method	We tested the solutions to 5 patients using a concurrent think-aloud process. We gathered verbal and visual feedback about the low-fidelity prototype from the patients, which we will use to build a high-fidelity prototype.		Interviews were conducted through Microsoft Teams by a moderator, HS, with a patient observed by other researchers (whose video and audio function was turned off). HS and each patient could see each other for an ice-breaking session at the start of the testing session. This session was essential to create rapport and to ensure the patient’s readiness, mentally and technically. The patient was then given a link to the prototype where they browsed through the prototype, gave comments and answered prompted questions. The patient’s screen was shared within the MS Team. The observers synchronously collected the patients’ replies on the utility and usability prompts about the prototype during the interview on the virtual scoreboard and the observer’s field notes. The moderator had accessed to the scoreboard and would be able to pick up any point which needed further clarification. The interviews were audio recorded, and browsing activities were video recorded.	

The web-based workshop discussions were conducted on a web conference platform (Microsoft Teams), and brainstorming of the idea was conducted on a web-based board (Miro). While conducting the exercise on the web-based board, the workshop participants remained connected to the web conference site to allow ongoing discussion. The web-based board was superseded by a prototype development (Figma) site in stages 4 and 5, whereas discussions remained on Microsoft Teams. The app developer supported information technology activities. Two weeks before the workshop, one of the researchers contacted each of the participants (patients and HCPs) to assess technical skills such as the ability to log on, use a meeting platform, and logistic issues such as the quality of the internet connection.

### Setting

The workshop, which took place between June 22 and 26, 2020, was conducted through a secure web-based meeting platform using a virtual whiteboard to facilitate information sharing between the researchers and app developers. Our original plan for a face-to-face workshop was changed to a web-based format to overcome the restriction of the order of movement because of the COVID-19 pandemic; an additional advantage was that it allowed participants from different locations and time zones to participate.

The patients were from 2 urban public primary care clinics in central Malaysia. Asthma is managed in primary care clinics, although chronic and acute care management through the provision of an asthma action plan to support asthma self-management is uncommon. Malaysia has a dual health system, public and private, where the public health system provides the leading service for the population with copayment of Ringgit Malaysia, RM 1 (US $0.23) per visit.

### Samples and Recruitment

#### Stakeholder Discussion (Stage 1)

A total of 3 patients and 2 HCPs who cared for asthma in the primary care settings, 2 app developers, 4 researchers from Universiti Putra Malaysia, and 1 from the University of Edinburgh were involved in the stakeholder discussion in stage 1 (Table 2).

**Table 2:** Summary of the stakeholders involved in each stage.

Stakeholder	Stage				
	1	2	3	4	5
1. Researchers	✓^a^	✓	✓	✓	✓
2. Patients	✓				✓
3. Health care professionals	✓				
4. App developers	✓	✓	✓	✓	✓

^a^Stakeholder present.

#### Testing of the Prototype (Stage 5)

Five patients, recruited from the Klang Asthma Cohort, participated in testing the prototype at stage 5 (Table 2). The Klang Asthma Cohort database is one of the research outputs of RESPIRE (National Institute for Health Research Global Health Research Unit on Respiratory Health) in Malaysia. The database contains 1280 people with asthma recruited from primary health care clinics in the Klang district. They provided consent to be called with invitations to participate in asthma-related research. Inclusion criteria for patients invited for this study were physician-diagnosed asthma, aged >18 years, smartphone user, limited health literacy, and were assessed by screening using the *Bahasa Malaysia* language version of the Health Literacy-Q47 scale [30]. The initial Health Literacy-Q47 scale [31] was translated and validated in Malaysia with a Cronbach α=.96 [30].

### Data Collection

Demographic information was collected from the database of patients who agreed to participate. Stakeholders’ discussions in stage 1 focused on challenges by patients and HCPs around (1) asthma education, (2) asthma self-management, (3) monitoring of symptoms, (4) emotional support or lifestyle advice, (5) social support, and (6) clinic set up (Multimedia Appendix 1).

In stage 5, we tested the prototype with 5 patients to assess its utility and usability using a set of semistructured questions in a concurrent think-aloud manner (Multimedia Appendix 2). During this session, HS, the main interviewer, guided the process. Four other researchers, PYL, SSG, ATC, and JW, observed the interview while the 2 app developers, Aidil Goh and Muhammad Marzuqi, managed the technical aspects of the sessions. The interviews took 1 hour and were conducted in *Bahasa Malaysia*, the patients’ preferred language.

Qualitative interviews were audio recorded, other web-based discussions and browsing activities were video recorded, and web-based board exercises were captured and archived as described in Table 1. All interviews were transcribed verbatim.

### Data Analysis

For this qualitative study, we used thematic analysis to obtain rich data from the stakeholders’ discussions in stage 1 and the interviews in stage 5. The texts were analyzed iteratively using a deductive thematic analysis approach, as outlined by Braun et al [32]. The deductive thematic analysis seeks to answer the researcher’s theory or analytical interest within the topic [32]. Phases in the thematic analysis included [32] (1) familiarization with the data by reading and rereading and noting down initial ideas (memoing), (2) duplicate coding (HS and JW) of one interview and comparing decisions to agree on standardizing the coding framework before coding all the transcripts, (3) discussing emerging themes with the research team, (4) reviewing themes with the wider research team and generating a map of the analysis (HP, SSG, PYL, ATC, or IY), (5) defining themes iteratively, and (6) presenting the deductive analysis with a selection of extracts. The data were organized using NVivo 11 (QSR International) qualitative data analysis software (HP and IY).

## Results

### Participants’ Characteristics

Five patients (including 3 who attended stage 1) attended stage 5 (prototype testing). Table 3 summarizes the demographics of the patients involved in stage 5.

**Table 3:** Patients’ demographic involved in stage 5.

ID	Age (years)	Gender	Education level	Health literacy score^a^	Use of pictorial asthma action plan at 6 months	Access to a digital device
P1	44	Female	Secondary	30	Yes	Smartphone and PC
P2	36	Male	Tertiary	32	No	Smartphone
P3	40	Female	Tertiary	17	Yes	Smartphone and PC
P4	38	Male	Tertiary	21	No	Smartphone
P5	19	Male	Secondary	31	No	Smartphone and PC

^a^Score less than 33 is considered as limited health literacy.

### The Outcomes of Each Stage

#### Stage 1: Understanding and Mapping Problems

Overall, 3 patients and 2 HCPs (a family physician and a medical officer from Klang district) contributed to the stakeholder’s discussion. The stakeholder discussion themes were categorized as relating to individuals, family and friends, society, and systems (Multimedia Appendix 3). The key problem used to inform the app design was education sources for asthma and support in the community, enabling self-management using pictorial action plans, reminders for medications, and asthma reviews or appointments. The log of history of asthma control, preventer intake, and information on expected best peak expiratory flow rate were features that could support patients during asthma review or appointment to discuss with their HCPs.

#### Stage 2-4: Sketching, Designing, and Developing the Prototype

Informed by the stakeholder discussion and findings of our previous qualitative study, we worked through the stages of sketching solutions and designed and developed the prototype (Multimedia Appendix 4). Through a round of voting in stage 3, solutions supporting self-management based on evidence-based practice were selected [3]. All winning solutions were clustered around four aspects of care: (1) education, (2) supporting self-management, (3) supporting behavior change, and (4) social support (Table 4). We created a storyboard for the prototype, and we developed the prototype app based on the storyboard, which was then tested in stage 5.

**Table 4:** Asthma App content and design features.

Main theme and section		Content	Features
**Education**			
	About asthma, its symptoms and diagnosis; asthma medications	Information in text and videos about asthma, symptoms, triggers, how the diagnosis is made, exacerbations and myths around asthma attack. Other information includes types of medications used to treat asthma, its function, and potential side-effects of the medications. There will be video-based instructions on the inhaler technique.	The links to the Ministry of Health portal on asthma was provided under specific headings to facilitate the search for reliable information.
**Supporting self-management**			
	Self-monitoring of symptoms	Patients indicate any experience of asthma symptoms in the last 24 hours, which will translate into control and prompts to check the action plan.	Tick-box list of potential asthma symptoms; ticking any one symptom will prompt a pop-up on advice to look at an action plan with a click button.
	Asthma action plan	A pictorial asthma action plan was used. Illustrations and wordings were validated in a series of discussions with stakeholders.	List of zones are displayed, and patient choose which zone are appropriate for them.
**Supporting behavior change**			
	Asthma medication and appointment reminder	Patients provide information about medications and appointments which will trigger a reminder system at the timing of choice.	Matrix of images of medications used and drop-down menu for frequency and timing
	Asthma diary	Asthma control and medication uptake will be recorded in the diary, including best PEFR^a^.	Monthly calendar, which displays asthma control and adherence
	Reward system	Achieving good asthma control and medication adherence will be translated into points.	Display of scale of points achieved for good asthma control and adherence
**Others**			
	Social support	Information regarding support groups for asthma in Malaysia	The links to various support groups available in Malaysia

^a^PEFR: peak expiratory flow rate.

**Figure 1:**
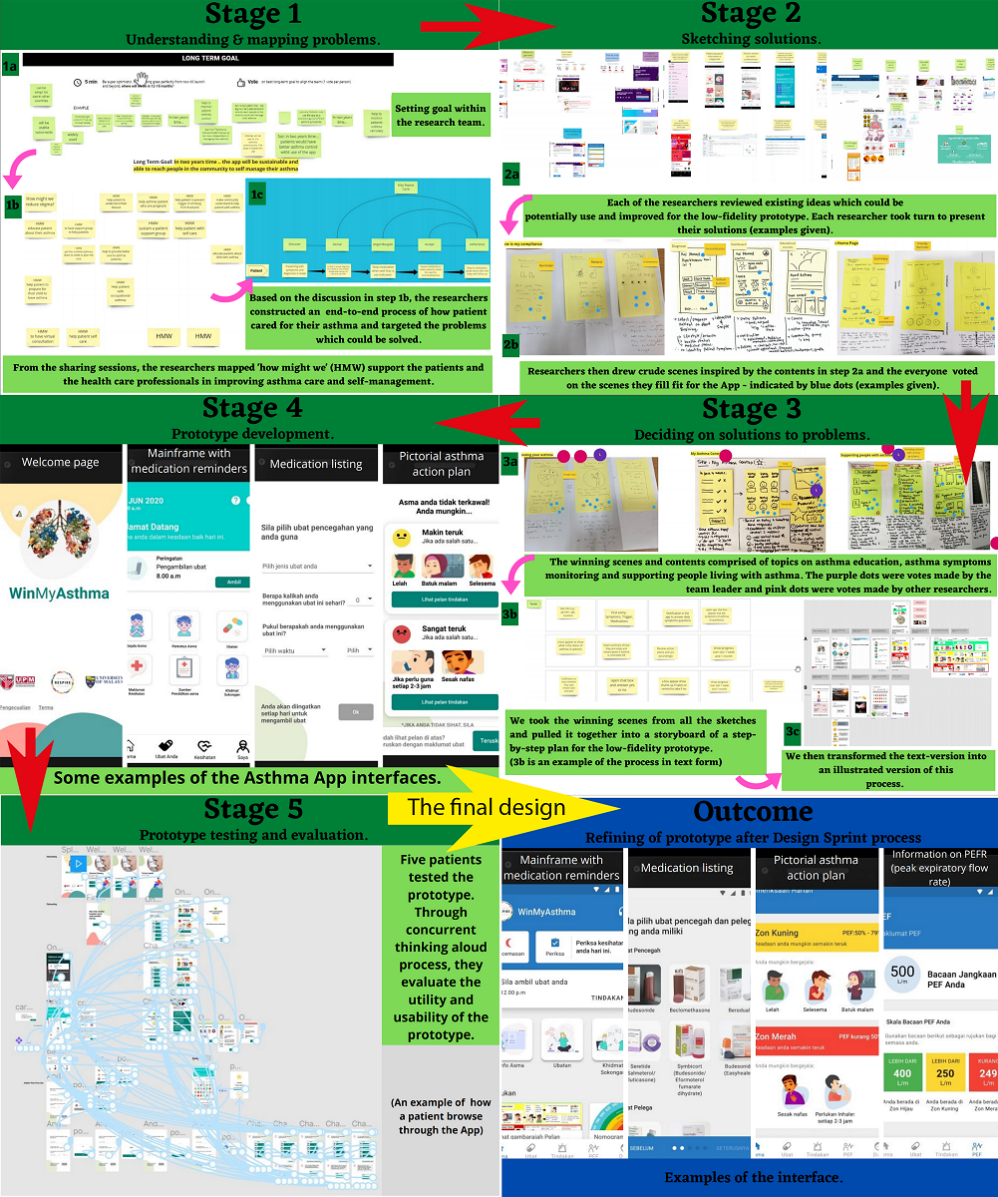
The Design Sprint process was undertaken on the web-based discussion board and meeting platform.

The entire process is illustrated in Figure 1, and the decisions on the prototype content are summarized in Table 4. The app was written in the Malay language.

#### Stage 5: Testing Low-Fidelity Prototype

Patients who attended stage 1, along with 2 other patients, attended stage 5. Results are presented under the 2 main themes of utility (ie, app influence or support self-management) and usability (ease of use).

##### Utility

Patients commented that the app influenced the decision to self-manage in four ways: offering information, providing an accessible asthma action plan, motivating and supporting improved medication adherence, and promoting behavior change through a reward system.

###### Offering Information

Patients considered that the app provided essential information regarding asthma and how to manage it. P4 explained, “the information about asthma in the App is interesting and informative.” Although many were comfortable reading text-based information, some preferred audio-visual formats such as videos. P5 explained, “the information about asthma, maybe it can be in the video, it’s more interesting than just text.”

###### Providing Accessible Asthma Action Plans

Patients felt that having an action plan on the phone made the plan accessible when needed. As P1 described, “when you need the plan, you just open the App in your phone and click on the plan [action], to see it.” In this format, patients considered it easier to access and use the app-based plan than the paper-based action plan, which they may not carry unless they attend medical appointments.

###### Motivating and Supporting Improved Medication Adherence

The medication reminder function of the app was viewed as a good support for achieving good adherence to daily preventers. P5 stated, “It will be difficult not to remember taking the medications because of the reminder, and because I use the phone frequently, it is hard to ignore the reminder (chuckled).”

###### Promoting Behavior Change Through a Reward System

The app was designed to encourage behavior change through a positive reward system, where good asthma control and adherence to twice daily preventive inhalers would be awarded points, and the cumulative points were visualized clearly. Patients liked this approach. P1 was incredibly excited to see the reward points on the app: “wow, there is a reward points, this is great!” This excitement was shared with other patients who preferred to see the tangible results of their actions. P3 elaborated on how the reward system could influence behavior:

That’s nice when I get points for taking the medications. I do want to see that I accumulate points and the scale moved further. And I can only do this if my control is good and if I take my medications.

##### Usability

The patients could easily comprehend the information and instructions in the app. P2 elaborated on how he achieved this: “the instructions are accompanied with illustrations. It makes it easier to understand it.” Although patients liked the use of illustrations and fewer words, the small font size used was challenging for some. P1 said:

[The] writing is small. I tried to put on my glasses, I still can’t see it (chuckled).

We used cartoon-based illustrations of the medications and in the steps of the asthma action plan. Some studies have suggested that the pictorial asthma action plan may be useful for patients with asthma other than adults. P3 suggested:

I think the illustrated plan can be appealing and useful for children and their carer. It’s very easy to understand.

Patients pointed out that a lack of navigational symbols meant that it was not always clear how to move from one interface to another. P4 stated his confusion:

There is no sign or indication on what to do next. I was a bit lost on what should I do now. Perhaps an arrow would help to tell that I can move forward.

Otherwise, patients were mostly satisfied with the simple layout. P1 gave an example of this:

In terms of the layout, it’s quite easy to navigate around the App. It’s ok for me.

The time spent using the app varied between 10 and 45 minutes. Some (younger) patients seemed comfortable navigating from one interface to the other and were keen to click buttons to explore the app functions. In contrast, others were dependent on symbols or prompted to navigate, which the app lacked at the testing stage.

The language used in the app was generally satisfactory, although there was a linguistic misunderstanding of *breathlessness* and *wheeze* in the *Bahasa Malaysia* language. P4 described his confusion on the *Bahasa Malaysia* words for breathlessness and wheeze:

Mengalami sesak nafas (breathlessness) and lelah (wheezing), are different? I thought it’s the same thing?

##### After the Workshop

**Figure 2:**
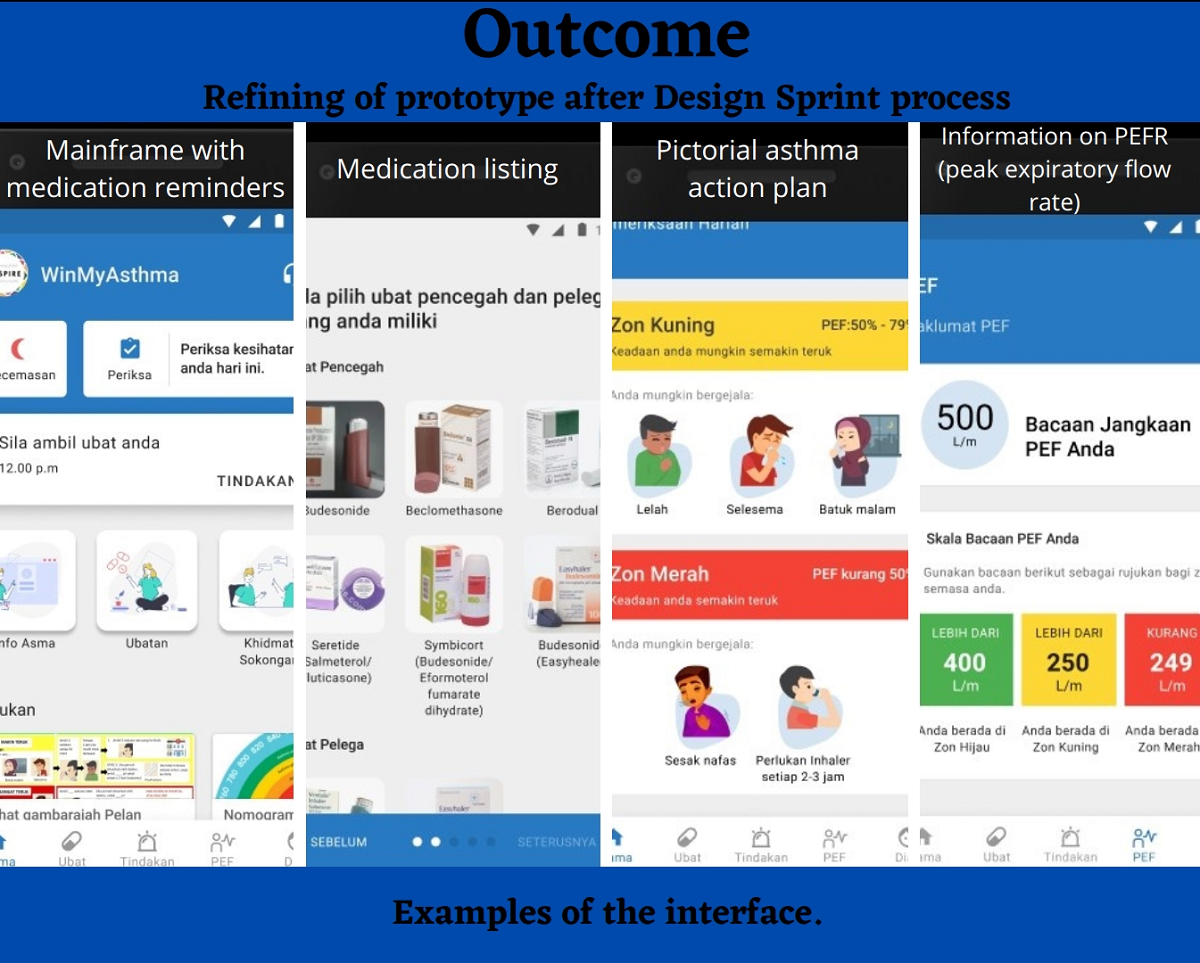
The finalized app design.

The design was refined and finalized after testing in stage 5 (Figure 2).

## Discussion

### Principal Findings

We conducted a design sprint workshop and employed a web-based format to ensure the safety of researchers and patients during the global pandemic. In this 5-stage workshop, we developed a low-fidelity prototype based on theoretical frameworks and refined it based on patient feedback during the design and testing stages. Patients described the resultant app to influence their ability to self-manage in four ways: offering information, providing accessible asthma action plans, motivating and supporting improved medication adherence, and promoting behavior change through a reward system. Specific usability issues were related to navigation, comprehension, and layout.

### Strengths and Limitations

The involvement of stakeholders and the multidisciplinary approach at the development stage in the design sprint process are among the strengths of this study, which may increase the chances of the intervention meeting the needs of the target population. The 5-stage design sprint structure allowed the development and testing process to be performed quickly and efficiently at a low cost, which would likely be favorable in low-resource settings. Constructing a low-fidelity prototype offers many advantages in the initial stages of prototype development. It allows a quick gathering of requirements, ideas, and concepts and can be built rapidly [29]. The disadvantage was that the low-fidelity prototype lacked some core functionality (such as navigation features), so that the patients on day 5 gave feedback on a limited version of the app. Nevertheless, the feedback was beneficial and enabled the app to be refined after the workshop to produce a high-fidelity prototype.

The web-based approach connected people in different geographical locations and ensured safety during the global pandemic. We recognized that we do not have any participants aged >50 years, perhaps because this approach may be more appealing to younger age groups, although with limited health literacy, and it may not reflect the feasibility of using web-based methods for older age groups. We overcame limited internet access by providing an internet data voucher; however, we had to exclude those with no access to any digital devices. We provided training on the various platforms to be used in the workshop, thus overcoming the lack of digital skills. These strategies may assure researchers or intervention developers working in countries or settings with high levels of limited health literacy that this web-based methodology is of value.

The pandemic context may have explained the relatively small number of participants recruited during the testing stage. Some participants found it challenging to commit uninterrupted time to a web-based workshop while being *locked down* at home with their domestic or caring or *home-schooling* responsibilities. For comparison, face-to-face intervention design workshops have been reported with 14 participants in 5-day workshops [33] and 38 participants over 6 weeks [34].

### Adaptation of the Design Sprint Workshop to a Web-Based Format

Patients with asthma and HCPs were recruited for a workshop in March 2020, but this could not occur because of the compulsory lockdown imposed by the local authorities in response to the COVID-19 pandemic. Therefore, we adapted the workshop to the challenges of a web-based format and its potential impact on participants’ research experience [35]. A high-speed internet connection and technical skills in conducting this workshop were vital. To overcome some of these practical issues, we supported all participants with mobile web-based access through an RM 10 (US $2.42) internet data voucher to ensure that the participants would not bear the cost of internet access. Participants’ experience with technologies was around social media, that is Facebook, video call, that is, WhatsApp, and information searching platforms, that is, Google and YouTube. We found a lack of experience with videoconferencing platforms and the software we planned to use during the testing day. Thus, we conducted training sessions for all participants to avoid technical problems during the workshop.

In the context of interviews, the literature suggests that web-based data collection can produce data of similar quality to face-to-face interviews [35]. Although using a web-based platform to interview patients was a new experience for the researchers, we found that the web-based programs eased discussions, and interviews took place quickly and effectively. Compared with traditional qualitative interviews, one advantage was that other researchers could observe the interview sessions on the web-based platform, and they could make concurrent fieldnotes. From the patient’s feedback, although they knew they were being observed by additional researchers, being at their own home helped them forget about being observed and anxious.

In web-based discussions and interviews, dictation software has been used to capture audio data in text format, thereby avoiding transcription errors [36,37]. However, this was not possible in our context because the medium of interaction was the *Bahasa Malaysia* language, and the extensive use of colloquial language made it impossible to use any dictation software. Conducting research on the web raises concerns about participants’ confidentiality and data security. We ensured that entry to the workshop was password-protected to control access to maintain the participant’s confidentiality securely. The recordings were stored in a secure manner. For example, files from the workshop were encrypted and stored in a secure research data storage facility.

### Web-Based Design Workshop and the Context of Limited Health Literacy in a Low-to-Middle–Income Country

Our app focused on designing a pictorial asthma action plan as a core strand of tailoring supported self-management for people with limited health literacy. Other features were a simple language for symptom assessment, education and information resources, provision of visual and audio medication reminders, and practical behavior change strategies such as a reward system. A clear message from our previous qualitative work was that participants wanted an interactive approach to support an asthma action plan with few words and clear pictures. Our original plan was to provide a paper-based pictorial action plan, but as it is not interactive, we reconsidered potential formats and decided to deliver the pictorial asthma action plan using a mobile app. Using a mobile app was seen as promoting a sense of autonomy to feel empowered in managing asthma.

Although previous reviews have reported a lack of interest in action plans [21], others have reported on the keenness of people to use action plans in mobile apps [38], although none have explored plans tailored for people with limited health literacy or the innovative pictorial representation of actions in a mobile app. In the United States, a study found that action plans were written at the literacy level of sixth-grade (11- to 12-year-olds), which will be a challenge to those without formal education or only receiving primary school education [39]. The same study also found that more graphics within an action plan may be needed to increase the ease of use [39]. In our web-based design workshop, the extensive use of images, icons, and the use of simple language were among the strategies used to overcome the challenge of understanding a written asthma action plan.

### Recommendations for Practice, Policy, and Research

We outlined recommendations for practice, policy, and research based on this study in Textbox 1. On a practical level, to help researchers’ concentration throughout the 5 days of the 7-hour workshops, we included frequent breaks and provided high-energy snacks. Each session was either 1 hour long with a 15-minute break or 45 minutes long with a 10-minute break. Committing to time in a workshop alone in front of the monitor can be challenging and mentally draining, so a week before the workshop, each researcher received a supply of high-energy snacks through the post. We also provided colored sticky notepads and permanent markers with similar tip sizes to ensure that all scanned sketches and writings were clear when uploaded on the web-based discussion board.

**Recommendations for practice, policy, and research.**

**Practice**

- Although some participants only join the workshop for short periods, researchers and technical colleagues have to concentrate on the web for long periods. Adequate breaks, attention to nutrition, and general comfort are essential.
- The web-based platforms may be unfamiliar to many participants; training before the workshop gives confidence and helps reduce technical problems on the day.
- Owing to the relatively low cost and a short time spent from development to testing, the web-based design sprint methodology may be suitable for low-resource settings.
- Remote conduct ensured that high-risk stakeholders were shielded during a pandemic and overcame geographical barriers.

**Policy**

- The process is *a sprint* so that the end product can be developed to a short timescale to meet pressing deadlines.

**Research**

- The feasibility study of conducting a more extensive scale web-based intervention design program is necessary to ensure its practicality.

### Conclusions

Working with people with limited health literacy enabled the development of an app that could support them in self-managing their asthma. Specific components included sources of information on asthma, pictorial asthma action plan, simple language, audio-visual prompts, and rewards for supporting adherence to daily therapy and scheduled reviews. Despite practical challenges, a 5-day web-based design workshop proved to be manageable, enabling meaningful engagement from patients and HCPs so that a prototype is now ready for feasibility testing.

## Appendix

Multimedia Appendix 1 Prompts for stakeholder discussion.

Multimedia Appendix 2 Utility and usability prompts.

Multimedia Appendix 3 Themes from the stakeholders' discussion.

Multimedia Appendix 4 Stages 2-4.

## Abbreviations

HCP: health care professional
RESPIRE: National Institute for Health Research Global Health Research Unit on Respiratory Health
