# Supplementary material for: Developing an Asthma Self-management Intervention Through a Web-Based Design Workshop for People With Limited Health Literacy: User-Centered Design Approach
Source: J Med Internet Res. 2021 Sep 9;23(9):e26434. doi: 10.2196/26434 (PMC8461531; doi:10.2196/26434)
Supplement: Multimedia Appendix 1 [file jmir_v23i9e26434_app1.docx]

Appendix 1 Prompts for stakeholder discussion.

1. How can asthma self-management be enabled?
2. How can asthma symptoms be better monitored?
3. How can delivery of education on asthma be improved?
4. What is the support other than healthcare which patient needs?
5. How healthcare professionals can be supported to improve asthma care?
