# Supplementary material for: Developing an Asthma Self-management Intervention Through a Web-Based Design Workshop for People With Limited Health Literacy: User-Centered Design Approach
Source: J Med Internet Res. 2021 Sep 9;23(9):e26434. doi: 10.2196/26434 (PMC8461531; doi:10.2196/26434)
Supplement: Multimedia Appendix 2 [file jmir_v23i9e26434_app2.docx]

Appendix 2 Utility and usability prompts.

**Utility questions**

Q1: Is the information in this App useful?

Q2: Based on the information in this application, do you know what to do if you have asthma symptoms?

Q3: Does the information in this App help you to take your daily medications?

Q4: Will the information in this App motivate you to control asthma?

Q5: Would you recommend this application to someone with asthma?

**Usability questions**

Q6: Do you understand the information displayed in this application?

Q7: Do you understand the language used in this application?

Q8: Is it easy for you to browse this application?

Q9: Is the layout in this application appropriate?

Q10: Is the illustrations or pictures in this App interesting?

Q11: Will you continue to use this application?
