# Supplementary material for: Developing an Asthma Self-management Intervention Through a Web-Based Design Workshop for People With Limited Health Literacy: User-Centered Design Approach
Source: J Med Internet Res. 2021 Sep 9;23(9):e26434. doi: 10.2196/26434 (PMC8461531; doi:10.2196/26434)
Supplement: Multimedia Appendix 3 [file jmir_v23i9e26434_app3.docx]

Appendix 3 Themes from the stakeholders' discussion

| Level | ASTHMA SELF-MANAGEMENT | ASTHMA MONITORING | EDUCATION | EMOTIONAL SUPPORT/LIFESTYLE ADVICE | SOCIAL SUPPORT | CLINIC SET-UP |
| --- | --- | --- | --- | --- | --- | --- |
|  | **HOW MIGHT WE…..** | | | | | |
| Individual level | help patient remember their appointment and adherence to medications using technology? | Help patients to monitor their symptoms? | Help patients identify their triggers? | Motivate patients to continuously engage with asthma care? | Create awareness about social support for people with asthma living in the community? |  |
|  | Assist patients on how to act during exacerbations? | assists patients to manage their symptoms in interactive manner? i.e. live chat with health professionals, teleconsultation | Deliver knowledge about asthma and its control effectively to patients? | Assists patients to cope with their activities of daily living? |  |  |
| Family and friends |  |  | Create and improve family awareness about the potential serious impact of poorly controlled asthma? | Effectively engage family members in asthma care? | Assist patients to get better social support from peers and social network? |  |
| Society |  |  | Create and improve public awareness about the potential serious impact of poorly controlled asthma? |  | Assist patients to get better social support available in the society? |  |
| System level |  | Reduce clinician's workload in asthma care? |  |  |  | Assist clinicians to have better knowledge on source of support for patients? |
|  |  | Provide clinician with asthma control assessment to monitor patients? |  |  |  |  |
|  |  | Create monitoring system that could provide advice which corresponds to current situation? i.e. COVID-19, haze |  |  |  | Create an App to reduce clinician workload? |
|  |  |  |  |  |  | Provide dedicated asthma clinic for all patients? |
|  |  |  |  |  |  | Provide a multidisciplinary care? |
