# Supplementary material for: Developing an Asthma Self-management Intervention Through a Web-Based Design Workshop for People With Limited Health Literacy: User-Centered Design Approach
Source: J Med Internet Res. 2021 Sep 9;23(9):e26434. doi: 10.2196/26434 (PMC8461531; doi:10.2196/26434)
Supplement: Multimedia Appendix 4 [file jmir_v23i9e26434_app4.docx]

Appendix 4: Stages 2-4

| Stages | Solutions gathered from expert discussions. (Expert includes literature, previous studies and stakeholder discussions) | |
| --- | --- | --- |
| Stage 2  Informed by the the outcome of Stgae 1, brain-storming of solutions took place at the end of stage 2. |  |  |
| Stage 3  The research team voted on best solutions based on evidence-based practice. |  |  |
| Stage 4  Winning solutions were clustered based on the App sections. |  |  |

*Footnotes: The bubbles of topics were consolidated in each stage through voting process.*
